# Functional Near-Infrared Spectroscopy and Its Clinical Application in the Field of Neuroscience: Advances and Future Directions

**DOI:** 10.3389/fnins.2020.00724

**Published:** 2020-07-09

**Authors:** Wei-Liang Chen, Julie Wagner, Nicholas Heugel, Jeffrey Sugar, Yu-Wen Lee, Lisa Conant, Marsha Malloy, Joseph Heffernan, Brendan Quirk, Anthony Zinos, Scott A. Beardsley, Robert Prost, Harry T. Whelan

**Affiliations:** ^1^Department of Neurology, Medical College of Wisconsin, Milwaukee, WI, United States; ^2^Department of Neurology, Children’s Hospital of Wisconsin, Milwaukee, WI, United States; ^3^School of Medicine, University of Washington, Seattle, WA, United States; ^4^Department of Biochemical Engineering, Marquette University and Medical College of Wisconsin, Milwaukee, WI, United States

**Keywords:** functional NIRS, near-infrared spectroscopy, functional MRI, cytochrome c oxidase, epilepsy, migraine, autonomic dysfunction, cerebral autoregulation

## Abstract

Similar to functional magnetic resonance imaging (fMRI), functional near-infrared spectroscopy (fNIRS) detects the changes of hemoglobin species inside the brain, but via differences in optical absorption. Within the near-infrared spectrum, light can penetrate biological tissues and be absorbed by chromophores, such as oxyhemoglobin and deoxyhemoglobin. What makes fNIRS more advantageous is its portability and potential for long-term monitoring. This paper reviews the basic mechanisms of fNIRS and its current clinical applications, the limitations toward more widespread clinical usage of fNIRS, and current efforts to improve the temporal and spatial resolution of fNIRS toward robust clinical usage within subjects. Oligochannel fNIRS is adequate for estimating global cerebral function and it has become an important tool in the critical care setting for evaluating cerebral oxygenation and autoregulation in patients with stroke and traumatic brain injury. When it comes to a more sophisticated utilization, spatial and temporal resolution becomes critical. Multichannel NIRS has improved the spatial resolution of fNIRS for brain mapping in certain task modalities, such as language mapping. However, averaging and group analysis are currently required, limiting its clinical use for monitoring and real-time event detection in individual subjects. Advances in signal processing have moved fNIRS toward individual clinical use for detecting certain types of seizures, assessing autonomic function and cortical spreading depression. However, its lack of accuracy and precision has been the major obstacle toward more sophisticated clinical use of fNIRS. The use of high-density whole head optode arrays, precise sensor locations relative to the head, anatomical co-registration, short-distance channels, and multi-dimensional signal processing can be combined to improve the sensitivity of fNIRS and increase its use as a wide-spread clinical tool for the robust assessment of brain function.

## Introduction

Functional near-infrared spectroscopy (fNIRS) is a well-established non-invasive tool to continuously assess regional tissue oxygenation at bed-side. It was first described by Jöbsis 40 years ago (Jobsis, 1977) and has been utilized in different clinical settings, especially in the field of neuroscience (Obrig, 2014; Hong and Yaqub, 2019). The current review aims to discuss the mechanism of fNIRS, its advantages and limitations in detecting brain activity, the current status of fNIRS clinical applications, and the future directions for developing fNIRS into a more widespread clinical tool.

The brain is a high energy-demand organ and neuronal activation correlates with increases in cerebral blood flow and volume. This so-called “neurovascular” coupling is the fundament of many functional neuroimaging techniques, including fNIRS, functional magnetic resonance imaging (fMRI), positron emission tomography (PET) and single-photon emission computerized tomography (SPECT). By measuring changes in the light absorption of different hemoglobin species, temporal changes in cerebral blood flow can be calculated with fNIRS. Several features of fNIRS, including portability, non-invasiveness, cost effectiveness and tolerability, make it an advantageous tool in both clinical care and neuroscience research. Concurrent monitoring of other real-time physiological parameters, such as EEG (Hong and Khan, 2017; Khan and Hong, 2017; Hong et al., 2018; Khan et al., 2018), can further enhance its temporal resolution, making it an ideal tool for the study of epilepsy, autonomic function, and physiologic phenomena.

Indeed, fNIRS has become a tool for standard care in pediatric ICUs to assess regional oxygenation, such as somatic (Balakrishnan et al., 2018) and cerebral oxygenation (Hoffman et al., 2017), in real-time. It is also widely used to assess task-related cortical function. By using a block design, regional cerebral blood flow signals can be enhanced to characterize task-based cortical function (see (Hong and Yaqub, 2019) for a comprehensive review). In this review we will focus on language mapping which has been the main interest of neurologists and neurosurgeons.

When it comes to more sophisticated measurement in real-time, fNIRS has encountered several limitations. First, oligochannel fNIRS used in most of clinical settings lacks spatial resolution which is crucial for functional localization in cognitive tests and source localization in seizure detection. Second, although the development of multichannel fNIRS greatly increases the spatial resolution of this technology, individual event analysis remains challenging due to the reduced signal-to-noise ratio (SNR) of fNIRS. Group analysis and signal processing can increase the SNR, but it is still unable to analyze individual events, such as seizures, in real-time. Techniques such as vector diagram analysis to detect the initial dip in the hemodynamic response have helped to tackle this problem (Zafar and Hong, 2018). Third, compared with EEG, the temporal resolution of fNIRS may be suboptimal to capture individual neurologic events. Seizures spread quickly between neurons on a millisecond timescale and can require a high sampling rate for accurate source localization (Coelli et al., 2019; Vespa et al., 2020). Sampling rates of clinical EEG are typically between 256 to 1024 Hz, whereas the sampling rate of fNIRS is an order of magnitude lower. Finally, reproducibility in the spatial localization of fNIRS is often limited by a lack of co-registration between individual subject’s anatomy and sensor placement. Co-registration of standardized optode placement to individual subject anatomy and individualized generation of forward models of fNIRS signal propagation, coupled with fMRI validation, could help address this issue.

With appropriate selection of light wavelengths, fNIRS characterization of neurovascular coupling can be expanded to also include metabolic function, for example using cytochrome c oxidase (CCO) (Bale et al., 2018; Holper and Mann, 2018; Lange et al., 2019). Coupled with the ability to simultaneously measure fNIRS together with other neuroimaging analysis techniques such as EEG and neural network learning (Yang et al., 2019) fNIRS has the potential for widespread clinical applications in neuroscience.

## Neurovascular Coupling

The lack of an energy storage capability in neurons means that increased metabolic activity associated with neuronal communication requires a ready supply of glucose and oxygen. Fox and Raichle (1984) demonstrated with PET that volitional tasks caused an increase in local glucose utilization as well as local oxygen utilization in the brain regions associated with the functional task. In the resting state, neurons have a relatively constant oxygen extraction fraction, resulting in a steady ratio of oxygenated to deoxygenated blood in the capillary bed surrounding the neurons. When going from a resting state to an active one, the local oxygen extraction fraction increases, which first increases the concentration of deoxyhemoglobin. Shortly after this begins, sphincters in the arterioles located just before the capillary bed dilate, flooding the capillaries with oxyhemoglobin. MRI detects this change as a change in the magnetic state of the blood. Oxyhemoglobin is diamagnetic, but when it releases its bound oxygen atoms to become deoxyhemoglobin, it becomes paramagnetic. The local change in the magnetic susceptibility of the tissues surrounding the capillary bed changes the T2^∗^ relaxation rate of the visible hydrogen nuclei. In the first 1–2 s of activation, the excess of deoxyhemoglobin decreases T2^∗^, causing the intensity of the voxel to decrease (initial dip). This phenomenon is not as reliably detected as the subsequent, and typically larger, increase in oxyhemoglobin concentration, which increases the T2^∗^ of the tissue and the corresponding intensity of voxels within the MR image.

Functional MRI, which measures changes in blood oxygenation/deoxygenation in the brain vis-à-vis the blood-oxygen-level-dependent (BOLD) response, has become a cornerstone of functional neuroimaging. While fMRI has been widely used to characterize neuronal activity in both healthy volunteers and patients, it has several limitations. The first is that the detection of brain activity is indirect. Because fMRI measures changes in blood oxygenation rather than neuronal activity directly, the effect can be extinguished or precluded by other vasoactive processes. A second important limitation is the need for a high-field-strength MRI system. The subject is confined in a magnet, and both the subject and any experimental apparatus must be magnet-safe. A final limitation is that the BOLD response measured with fMRI is based on the ratio of oxy- to deoxy-hemoglobin. These two moieties of hemoglobin are not individually detected.

Unlike fMRI, fNIRS measures the two hemoglobin species separately. The differentiation between hemoglobin species facilitates the use of differential analysis techniques, such as vector diagram analysis, using 2 (oxy- and deoxyhemoglobins) or 4 (oxy-, deoxyhemoglobin, the difference between them and the total hemoglobin) components to better define the initial dip in the hemodynamic response (Zafar and Hong, 2017, 2018). Moreover, fNIRS is portable, tolerable and cost effective. Timely bed-side assessment of cerebral oxygen can be performed more easily with fNIRS than fMRI. Functional NIRS is also a safe technology for patients with non-MR compatible implanted devices such as a vagal nerve stimulator (VNS), deep brain stimulator (DBS), or pacemaker.

## Mechanisms of fNIRS

Near-infrared spectroscopy in the brain is made possible by the relative transparency of biological tissues (including bone) to light for infrared wavelengths ranging from 650 to 925 nm. Light in this range is absorbed by oxyhemoglobin and deoxyhemoglobin much more strongly than surrounding and overlying tissues. The absorption of infrared light as a function of wavelength is different for these two chromophores, with deoxyhemoglobin absorbing more strongly below 790 nm and oxyhemoglobin more strongly above 790 nm.

**FIGURE 1:**
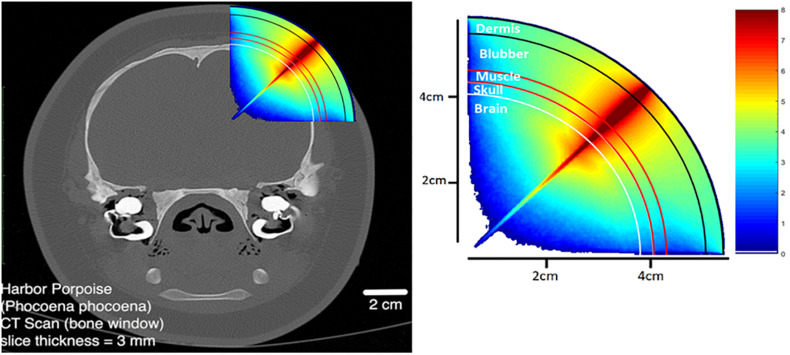
Monte Carlo photon propagation simulation using a point LED source incident at an angle normal to the scalp. Anatomy modeled on the harbor porpoise.

By detecting the changes in the relative concentrations of different light-absorbing molecules, fNIRS allows the analysis of energy metabolism in the brain. For example, fNIRS can measure changes of oxygenated and deoxygenated hemoglobin in a manner similar to fMRI, and thus reflect regional changes in neuronal activation. In order to do so, sufficient near-infrared light must be incident on the cortical surface. The attenuation of light by the skull, scalp and meninges can be predicted by Monte Carlo simulation (Hiraoka et al., 1993) as shown in Figure 1, to account for the static light-absorbing properties of the surrounding tissue.

**FIGURE 2:**
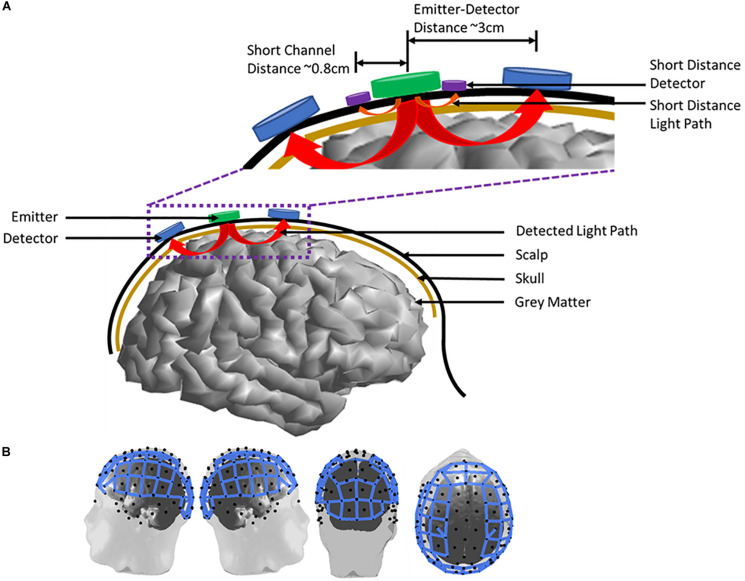
Emitter and detector arrangement on an adult human subject. **(A)** A two channel emitter-detector pair placement on the scalp. Arrows from the emitter to the detectors indicate the measured light path of each channel. The inset highlights emitter-detector distances and the incorporation of short-distance channels to measure scalp blood flow. **(B)** Full head imaging cap example on an adult human subject. Blue lines represent measurement channels (*n* = 102 channels) between emitter (*n* = 32) and detector (*n* = 32) pairs.

Commercial fNIRS systems utilize separate illumination sources and detectors. Typical source-detector separation is on the order of 1.5–3 cm in children and 2.5–5 cm in adults depending on head circumference, although it is recommended that separation does not exceed 3.5 cm in adults. In recent years, the use of short separation channels, <1 cm separation from source to detector, have been incorporated in studies to estimate and remove the blood flow contribution of the scalp in typical source-detector channels. Thus, models of light absorption by the surrounding tissues must also account for the path that the light takes to reach the cortex and be reflected back to the detector. This situation is shown in Figure 2.

## Noise Reduction and Signaling Processing

Multiple sources of signal interference (i.e., noise) can complicate interpretation of the fNIRS signal and has been a major challenge in clinical settings. Sources of noise can include head motion, variations in coupling over time due to changes in the distances between optodes (sources and detectors) on the scalp, and changes to blood flow unrelated to neuronal activity. Whereas fMRI measures the ratio of oxy- to deoxy-hemoglobin as a result of changes in bulk magnetic properties, fNIRS measures oxyhemoglobin and deoxyhemoglobin separately which can be confounded by changes in heart rate and blood flow. Since the near-infrared wavelengths must first pass through the meninges, skull, and scalp, physiologic changes in these tissues can induce changes in the light absorption between the source and detector that may not be related to functional changes in neuronal activity. Moreover, like fMRI, the response of the fNIRS signals to changes in neuronal activity are convolved with a hemodynamic response function which describes the latencies, overshoots and undershoots of the arteriole sphincters that control blood flow into the capillary beds in responses to changes in metabolic demand of the neuronal tissue (Osharina et al., 2010).

A number of signal processing techniques have been developed to separate task related signals from noise. The following sub-sections provide a brief overview of current approaches for reducing sources of physiological noise and motion artifacts.

### Reducing Physiologic Sources of Interference in fNIRS

The sources of physiological noise present in fNIRS include heart rate, blood pressure fluctuations, respiratory rate, and scalp blood flow. Several approaches have been used to identify and remove physiologic noise including digital filtering, prewhitening, adaptive filtering, data driven methods such as principle component analysis (PCA) and independent component analysis (ICA), and the use of short separation channels. Since different biological functions typically occur over different ranges of frequencies (Cordes et al., 2001; Blanco et al., 2018) digital filtering can be used to reduce or remove sources of interference that occur at temporal frequencies distinct from the task-related measures of brain activity (Cordes et al., 2001; Liu et al., 2017). However, blood pressure fluctuations (0.08–0.12 Hz) and resting heart rate (1–1.5 Hz) can overlap in frequency with the signals of interest and confound task-related signals presented in certain types of block designs (Huppert, 2016).

Another option is to remove physiological noise is through prewhitening. Prewhitening can be used to remove temporally autocorrelated signals, such as heart rate, by decorrelating physiologic signals not associated with the task (Barker et al., 2013). To determine the optimal prewhitening filter coefficients, Barker et al. (2013) and Blanco et al. (2018) used an iterative autoregressive model to reduce the residual error in the task-related activity estimated from a general linear model analysis. Prewhitening accuracy can be can be affected by motion artifacts (Blanco et al., 2018). Therefore, before prewhitening is applied any motion artifacts present in the signal should be removed (see section “Noise Reduction of Motion Artifacts” below).

Adaptive filtering techniques use a system of linear functions within either an open or closed loop control model. Nguyen et al. (2018) applied this principle to reduce the amount of physiological noise present in fNIRS during a finger tapping task through the linear combination of the expected hemodynamic responses to the prescribed stimuli, short separation channel signals to detect extra-cortical noise, Fourier approximations of physiological noise (heart rate, respiratory, and blood pressure fluctuations), and baseline drift. Unknown model coefficients were estimated using a recursive least-square estimator to produce an adaptive filter that was able to reduce on average 77% of noise in oxyhemoglobin and 99% of noise in deoxyhemoglobin (Nguyen et al., 2018). Some limitations of this type of filtering include parameter tuning, the need to define noise distributions, and biased estimations if the filter is not closed loop (Abdelnour and Huppert, 2009).

Changes in fNIRS due to global blood flow at the scalp can pose an additional signal confound especially in more demanding functional tasks. Spatial analyses, such as PCA, can be used to remove global blood flow not associated with a functional task (Zhang et al., 2016). PCA can be particularly effective when there is one dominant source of variation, such as either global blood flow or motion artifacts, but can fail if there are multiple sources driving the overall variation (Zhang et al., 2016). Furthermore, PCA approaches require multiple channels to reliably parse the global blood flow from physiologic signals of interest. Gaussian kernel approaches, similar to those used in fMRI, have also been shown to remove global blood flow at the scalp when applied spatially across channels (Zhang et al., 2016) although the ability to account for more localized spatial changes in blood flow produced by large vasculature is not well accounted for. Lastly, ICA (Hyvarinen and Oja, 2000), has been used to remove global blood flow during gait experiments by leveraging the temporal coherence between channels to identify large signal component(s) with a high coefficient of spatial uniformity (Kohno et al., 2007). While the use of ICA for artifact removal in EEG is well-established, its application to fNIRS has been limited.

More recently, short separation channels (∼8 mm source-detector distance) have been used to directly measure and remove scalp blood flow from fNIRS (Gagnon et al., 2014; Funane et al., 2015; Nguyen et al., 2018). The short distance between the light emitters and detectors prevents light penetration to the cortical surface, limiting blood flow measurements to the scalp. Funane et al. (2015) showed that hemoglobin signals obtained with short distances channels (∼1.5 cm) were better correlated with laser-doppler flowmetry measures of scalp blood flow than signals obtained from standard emitter/detector distances (∼3 cm) targeted to measurements of blood flow in adult cortex. Additionally, Nguyen et al. (2018) showed there was no correlation (*r* < 0.38) of short separation channels to other physiological noises present in long range separation channels. Thus, the inclusion of short separation channels as a regressor in fNIRS analyses can reduce signal interference from scalp blood flow.

### Noise Reduction of Motion Artifacts

Motion artifacts can occur from talking or movements of the face, head, and/or upper body (Izzetoglu et al., 2010; Jahani et al., 2018). Typically when these movements are made, the optodes on the scalp become displaced resulting in sharp high frequency displacements, slow wave drifts, or baseline shift in the fNIRS signal (Jahani et al., 2018). Various methods have been used to remove motion artifacts including wavelet-based filtering, spline interpolation, and Kalman filtering. Wavelets can be particularly efficient at removing motion artifacts due to their time-frequency localization properties. Wavelet-based methods decompose the fNIRS signal into wavelet coefficients and removes those that fall outside of a predefined distribution, like a Gaussian distribution, under the assumption that they are related to motion artifacts (Robertson et al., 2010; Molavi and Dumont, 2012). Molavi and Dumont (2012) reported a reduction in motion artifacts in fNIRS collected from infants after using wavelets. Although they are efficient at removing spike artifacts, wavelet-based methods can produce an added baseline shift into the data around the spike artifact. Additionally, if artifacts do not meet the threshold criteria, they can continue to corrupt the signal.

Spline interpolation methods model motion artifacts as a series of spline functions and subtracts them from the data (Scholkmann et al., 2010). Scholkmann et al. (2010) reported an average 89.8% decrease in root mean square error between NIRS signals before and after spline interpolation. Although they found a reduction in motion artifacts, residual high frequency spikes remained following the spline interpolation (Scholkmann et al., 2010). In a comparison study, Jahani et al. (2018) showed that combining spline interpolations with the Savitzky–Golay filter or a robust locally weighted regression smoothing (RLOESS) was able to correct for baseline shifts and high frequency spikes without introducing additional artifacts into the signal. Even though both methods, spline-Savitzky–Golay and spline-RLOESS, produced similar results with respect to artifact removal (i.e., mean square error between the true and estimated hemodynamic response functions: 0.44 ± 0.06 and 0.56 ± 0.08 for spline-Savitzky–Golay and spline-RLOESS respectfully), the spline-Savitzky–Golay method had significantly faster processing time (16 sec), versus the spline-RLOESS (1800 sec), for 51 channels (Jahani et al., 2018).

Kalman filtering recursively improves the estimate of a signal with additional information added over time. Izzetoglu et al. (2010) compared the effectiveness of a Kalman filter, versus an adaptive filter and Wiener filter, for removing motion artifacts in fNIRS data of 11 subjects performing various speed head movements. They found that the Kalman filter significantly increased the signal to noise ratio (SNR) of fNIRS signals (SNR = [6.63 8.51]) compared to the adaptive filter (SNR = [2.79 4.17]) while no statistical difference was found between the Kalman filter and the Wiener filter (SNR = [5.25 9.05]). This is important as the Kalman filter can be applied to real time analysis without the additional sensors required for adaptive filters and does not require fNIRS signals to be statistically stationarity as with a Wiener filter (Izzetoglu et al., 2010). However, caution is warranted when using the Kalman filter for motion artifact removal as the accumulation of error over time due to instabilities if the filter is not set up properly, non-modeled system dynamics, or non-linearities in the data can impact performance (Izzetoglu et al., 2010).

## Redox States of Cytochrome C Oxidase

Aside from physiologic signal confounds and motion artifacts present in most imaging measures of brain activity, measuring variations in the hemodynamic response via changes in oxyhemoglobin and deoxyhemoglobin has inherent limitations. It is not sensitive enough to determine the cerebral metabolic rate of oxygen (CMRO2). Hemoglobin provides information on cerebral circulation and intravascular oxygenation but its concentration does not reflect the tissue’s ability on oxygen utilization. On the other hand, mitochondria are responsible for most of immediate cellular oxygen metabolism. Hence, CMRO2 directly relates to mitochondrial function and could help identify the clinical significance of hemodynamic change. CMRO2 can be calculated by combining cerebral oxygenation and arterial oxygenation with cerebral blood flow (CBF), assuming the arteriovenous blood volume ratio is fixed (Verdecchia et al., 2013). However, it has proven challenging to measure cerebral oxygenation in many clinical settings due to its invasiveness. Therefore, researchers have been focusing on a third chromophore, cytochrome c oxidase (CCO), which is a key element in oxidative metabolism in mitochondria, the “power station” of neurons.

**FIGURE 3:**
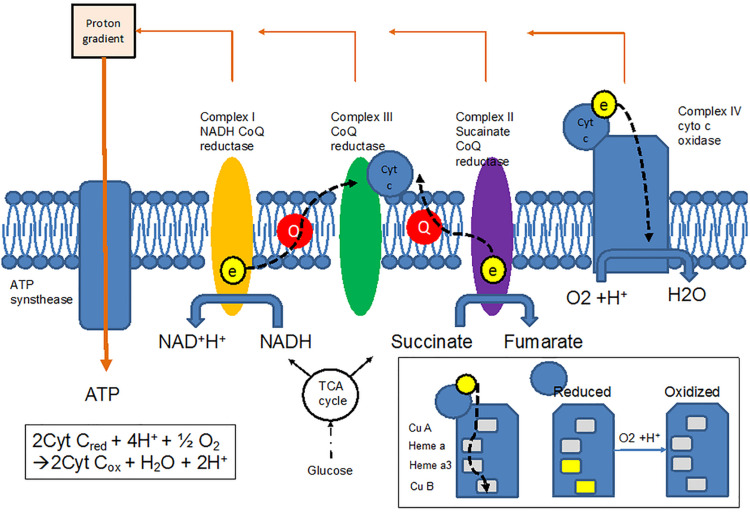
Electron transfer chain allows electrons to be transferred from the TCA cycle to oxygen via cytochrome c oxidase (CCO), resulting in changes in the redox states of CCO.

Cytochrome c oxidase plays a crucial role in the oxidative metabolism of glucose. Glycolysis metabolizes glucose into pyruvate in which the adenosine triphosphate (ATP) and nicotinamide adenine dinucleotide (NADH) are generated. The pyruvate is later transported into the mitochondria and converted to acetyl CoA. Acetyl CoA enters the tricarboxylic acid (TCA) cycle to generate more ATP and NADH. NADH is an electron donor in the electron transfer chain (ETC) (Figure 3); a series of protein complexes (known as complex I-V) that reside in the inner membrane of the mitochondria. Complexes I and II accept electrons from NADH and succinate, respectively, from the TCA cycle and transfer them to the soluble electron carrier coenzyme Q (CoQ). CoQ is oxidized by complex III, in the process the electrons used to reduce cytochrome c. Cytochrome c as a receiver for the electrons in the mitochondrial ETC is then oxidized by CCO (complex IV), which is a heme protein containing a binuclear copper center (CuA), a heme a, and a binuclear Fe-Cu center (heme a_3_-Fe) (Bale et al., 2016). The electron accepted from the cytochrome c is ultimately transferred to molecular oxygen, which is reduced to water. This process generates an electrochemical potential, which drives ATP synthesis via ATP synthase (complex V) in mitochondria. ATP is the ultimate energy source for cells.

All of these redox changes have associated optical transitions. In the range of near-infrared, the CuA center in CCO contributes the most to the absorption spectrum, around 830 to 840 nm. In theory, three wavelengths are required to simultaneously measure changes of CCO, oxyhemoglobin and deoxyhemoglobin. However, traditional continuous-wave fNIRS, which uses 2 to 3 wavelengths for hemoglobin species, is not suitable to quantify the change of CCO due to lower concentration of CCO and crosstalk between CCO, oxyhemoglobin, and deoxyhemoglobin in those wavelengths. Using multiple wavelength combinations is therefore important to accurately quantify the change of CCO and distinguish it from hemoglobin species. Although minimalistic approaches using fewer wavelengths are subject to noise and crosstalk, using too many wavelengths can be computationally prohibitive. Current advances in wavelength optimization suggest that with 8 wavelength combinations, the error rate for estimating changes of CCO can be reduced to less than 2% (Arifler et al., 2015). Redox changes in CCO may therefore allow fNIRS to detect changes in the electron transport of a heme protein, which absorbs light in the near-infrared spectrum and reflects mitochondrial energy metabolism.

Unlike hemoglobin species, CCO concentration does not change over a short time period.

The systemic fluctuation of blood pressure has little impact on CCO. Therefore, it is not influenced by the change of regional blood flow outside the brain as hemoglobins are. The redox state of CCO is not interfered by hemoglobin species and regional cerebral oxygenation (Springett et al., 2000). In theory, measuring the change of mitochondrial CCO better reflects cellular metabolism and viability. Potential clinical application of CCO will be further discussed in the next section.

## Clinical Application of fNIRS

### Language Mapping

Language is the most sophisticated high cortical function and the most important functional domain to localize during brain surgeries for tumor resection and epilepsy (Binder, 2011). Language mapping strongly correlates with the prognosis and outcomes of brain surgeries (Janecek et al., 2013b). Functional MRI has been the main tool for language mapping extra-operatively. It has high sensitivity but very low specificity (Rolinski et al., 2019). Therefore, invasive mapping such as direct cortical stimulation and Wada (intracarotid sodium amobarbital testing) test are inevitable in patients with bilateral or equivocal language representation in fMRI (Janecek et al., 2013a,b). Epileptologists, neurosurgeons and neuropsychologists have been looking for a non-invasive alternative and fNIRS has been studied for this purpose. Several lines of evidence have shown that fNIRS is a validated tool for neurocognitive functions (Hong and Yaqub, 2019). For example, research in human subjects suggests that the increase of prefrontal cerebral blood flow while performing cognitive tests can be detected by fNIRS (Masataka et al., 2015). Presurgical evaluations for refractory epilepsies have shown the reliability of fNIRS for language lateralization (Watanabe et al., 1998). Lateralization of inferior frontal activation during a written word-generation task was found to be consistent with handedness in a small study involving 11 healthy adults and 6 patients with medication-resistant epilepsy. The six patients with epilepsy had undergone the Wada test and showed the same lateralization for language as obtained with fNIRS (Watanabe et al., 1998). Another study using a category fluency task also suggested qualitative alignment of lateralization between fNIRS and either fMRI or Wada test results in small samples of adult and pediatric patients with epilepsy (Gallagher et al., 2007). Using a letter-fluency task administered to bilingual speakers in both Japanese and English, multichannel fNIRS showed additional areas of activation when performing the task in the later-acquired language, and this additional activation was correlated with second-language proficiency (Wroblewski et al., 2017).

Functional NIRS has its inherent limitations for language mapping and has not been used in a clinical setting. Limited temporal resolution and depth resolution of fNIRS pose the main obstacles for language mapping. Furthermore, fNIRS lacks anatomic specificity, which is crucial for surgical planning. Increasing the density of optodes and co-registration with other imaging modalities (e.g., MRI) could improve the anatomic specificity.

### Neurocritical Care

Anatomic specificity becomes less an issue in the setting of neurocritical care where timely measurements of global cerebral oxygenation is the main purpose. Functional NIRS has been widely used for this purpose given its non-invasiveness and good temporal resolution for hemodynamic changes. Several lines of evidence have shown that fNIRS has the potential to monitor cerebral hemodynamic change and cerebral autoregulation (CA) in the subacute phase of stroke. An early trial with a small number of patients (*n* = 10) revealed that the hypercapnia-induced increase in regional cerebral blood flow was impaired in the hemisphere affected by ischemic stroke (Vernieri et al., 1999). The authors concluded that dysfunctional CA played a pivotal role in the finding. A large-scale prospective trial (*n* = 98) suggests that dysfunctional CA correlated with delayed ischemic stroke after subarachnoid hemorrhage (SAH) (Budohoski et al., 2012). All patients had acute (<5 days) aneurysmal SAH and were treated according to the current clinical guidelines. Transcranial Doppler (TCD) was performed on each patient every 1–2 days to screen for vasospasm. Digital subtraction angiography was used to supplement the diagnosis. Bilateral frontal tissue oxygenation index (TOI) was obtained via cerebral fNIRS (NIRO-200). CA was calculated with both fNIRS and TCD as the linear correlation coefficient between arterial blood pressure (ABP) and TOI, and between ABP and middle cerebral artery flow velocity. The primary end point was cerebral ischemia within 21 days after SAH. In those who developed cerebral ischemia, TCD flow velocity failed to predict the development of cerebral ischemia while the autoregulation index showed changes 5 and 4 days before the ischemia, via fNIRS and TCD, respectively. This phenomenon suggests that fNIRS was able to demonstrate dysfunctional autoregulation 1 day earlier than TCD, which could make it a promising clinical tool for predicting the development of delayed ischemic stroke. Further research using continuous real-time fNIRS-generated CA indices might generate more clinically relevant utilization.

Frequency-domain fNIRS is another emerging tool for assessing CA in stroke patients. Low (LFO) and very-low frequency oscillation (VLFO) of oxyhemoglobin are found to be robust parameter for evaluating CA (Andersen et al., 2018; Su et al., 2018). Based on the cerebral oscillation theory, LFO and VLFO are associated with cerebral sympathetic activity. Furthermore, LFO reflects the myogenic component of CA; whereas VLFO reflects neurogenic (larger vasculature) and metabolic (microcirculation). Analysis of amplitudes and the interhemispheric synchronization of LFO and VLFO is a well-established method to assess the CA (see review (Andersen et al., 2018)). In patients with acute ischemic stroke (<5 days), the decrease in amplitudes of LFOs in the ischemic hemisphere and interhemispheric desynchronization were seen (Phillip et al., 2014). The effect of stroke on LFO and VLFO can last up to 12 months (Li et al., 2010).

Cerebral autoregulation is an increasingly recognized parameter in managing traumatic brain injury (TBI). Traditionally, intracranial pressure (ICP) and cerebral perfusion pressure (CPP) guide the pressure treatment in severe TBI. However, this concept is possibly oversimplified considering the individual differences between patients. Unlike ICP and CPP, CA is believed to be a more physiologic parameter and allows more flexible blood pressure management. Impaired CA results in secondary brain damage, which is an independent risk factor for fatality (Czosnyka et al., 2009). Therefore, autoregulation indices have been developed to identify the most optimal CPP (CPP_opt_) in the patient with severe TBI. The CPP_opt_ is determined by the correlation coefficient between the slow fluctuation in mean ABP and the ICP. This correlation coefficient is called the pressure reactivity index (PRx) in the field of neurocritical care, and a negative PRx is associated with favorable outcome. In a given patient, the CPP range in which the PRx is lowest is defined as “CPP_opt_.” Researchers have shown the correlation between the CPP_opt_ and patient morbidity and mortality (see review (Zeiler et al., 2017)). Typically, invasive ABP and ICP measurements are required to calculate PRx and optimal CPP, however, efforts have been made to find non-invasive alternatives to reliably calculate them (Dias et al., 2015; Bindra et al., 2016). For example, a recent prospective trial used multimodal brain monitoring, including ICP, CPP, bilateral transcranial cerebral oximetry with fNIRS, brain tissue oxygenation and cerebral blood flow, to assess the CPP_opt_ in patients with severe TBI. A total of 18 patients were included with a median Glasgow Coma Scale of 6. The CPP_opt_ was displayed every minute at bedside based on the CPP_opt_ curve calculated with invasive ABP and ICP data in the past 4 h. Therefore, the CPP_opt_ was monitored in virtual real-time for each patient, and the amount of time in which patients spent in impaired CA (PRx > 0.25) could be calculated. The ABP and ICP were managed based on the CPP_opt_ when it was available. Those who had high PRx and a longer period with PRx > 0.25 had statistically significantly poorer short-term and long-term outcomes. Further *post hoc* analysis suggested that PRx generated by transcranial cerebral oximetry with fNIRS showed the best degree of agreement with optimal CPP calculated by invasive PRx (Dias et al., 2015). These findings indicate that fNIRS can reliably supplement, or even replace, invasive ICP in terms of calculating CPP_opt_. Another trial of 19 patients with different critical neurological conditions including TBI used a similar approach to compare the validity of invasive and non-invasive Finometer photoplethysmograph ABP in calculating PRx (Bindra et al., 2016). This trial suggests that PRx can be obtained via an entirely non-invasive method (Finometer and fNIRS) in patients with critical neurological pathologies.

Oxygen saturation typically cannot be reliably estimated by continuous-wave fNIRS due to the unknown scattering coefficients in each patient. New fNIRS technologies allow researchers to assess the concentrations of hemoglobin species, rather than changes in them. For example, time-domain fNIRS has been used to investigate the cerebral oxygenation in patients with ischemic stroke (Giacalone et al., 2019). The result shows increased total hemoglobin but decreased oxyhemoglobin (namely, decreased oxygen saturation) in the ischemic hemisphere of large vessel stroke. Furthermore, a new approach combining measurements for hemoglobin species and CCO with time-domain fNIRS (Lange et al., 2019) can substantially enhance our understanding of dynamic neuronal oxygen metabolism in different cerebral pathologies. The non-invasiveness and the ability to measure the real-time physiological changes of fNIRS can also inspire researchers to further explore its potential for different clinical settings. Seizure detection is one of the most studied applications.

### Epilepsy

In the past decades, animal studies suggest that increased cerebral blood flow and oxygenation corresponding to epileptic spikes and high-frequency oscillation can be detected by fNIRS (Hoshi et al., 2001; Zhang et al., 2014). These changes identified by fNIRS can precede the appearance of spikes induced by bicuculline methiodide by about 5 s (Osharina et al., 2010). These findings have inspired many subsequent clinical studies in the field of epilepsy. The oxygenation response to seizures has been investigated extensively. There seem to be differences between focal and generalized seizure. For example, an early clinical case study revealed consistent ictal increase of oxygenation on the epileptic hemisphere relative to the non-epileptic hemisphere in patients with temporal and extratemporal lobe epilepsies (Monrad et al., 2015). This pilot study sheds light on further understandings about metabolic and hemodynamic change during seizure, such as uncoupling of oxidative phosphorylation, or alterations in cerebrovascular autoregulation. Other groups also suggest similar findings in focal-onset seizures (Villringer et al., 1994; Adelson et al., 1999; Sokol et al., 2000). In generalized epilepsies and secondary generalized seizures, regional frontal oxygenation has been shown to decrease during seizure (Sokol et al., 2000; Buchheim et al., 2004). Despite the small sample size of the studies (*n* = 2–8) and the use of oligo-channel fNIRS (1–2 channels), these preliminary findings paved the way for later larger-scale multi-channel trials.

Unlike other applications of fNIRS, precise localization and spatial resolution are crucial in epilepsy care and management. One of the current advances in fNIRS research is the development of multi-channel fNIRS devices. Multi-channel fNIRS is aimed at increasing spatial resolution, which has been the major obstacle of oligo-channel fNIRS. In the past decades, several case reports or series (*n* = 1–4) have been published. Differential increase in regional oxygenation or blood flow has been noted in SMA seizures (Sato et al., 2013), mesial temporal epilepsy (Rizki et al., 2015) and other focal seizures (Yucel et al., 2014). Utilizing multichannel fNIRS together with long-term EEG would allow epileptologists to better understand the hemodynamic changes during different phases of seizures (preictal, ictal, postictal and interictal phases) and improve seizure detection and localization. However, co-registering EEG, fNIRS or even MRI data requires advanced experimental skill sets and signal processing techniques to obtain and fuse multimodal imaging datasets. For example, placing multiple optodes and standard EEG electrodes simultaneously can be challenging for both patients and the electrodiagnostic technologists. Specialized software capable of displaying fNIRS and EEG recordings in real time is not yet commercially available. Analyzing small changes of hemoglobin species in the context of significant noise in fNIRS can be extremely challenging. The significant difference of sampling rates between fNIRS (tens of Hz) and EEG (∼500–2000 Hz) requires sophisticated mathematical techniques and computer power to temporally synchronize the recordings.

One of the pioneer trials, with a total of three presurgical patients, has overcome some of the technical issues (Nguyen et al., 2012). The customized electrode-optode combined cap allowed more efficient and comfortable placement of the sensors. The authors used a low frequency filter to minimize the cardiac artifact and a simple statistic tool (student’s *t*-test) to differentiate significant signal changes from the background. Selected fNIRS channels were averaged during the seizures to assess the overall variation. The authors found that total hemoglobin and oxyhemoglobin increased in the epileptogenic zone during the seizure, and deoxyhemoglobin exhibited a biphasic response with an initial decrement and later increment in the concentration. However, the lack of trials with larger patient numbers prevent the generalization of this practice. Furthermore, this trial did not show that the fNIRS provided additional localization value for seizure management. Indeed, one of the current clinical trials using portable multichannel fNIRS in 15 epileptic patients failed to show a significant seizure detection rate (Jeppesen et al., 2015). Only 6–18% of a total of 34 focal seizures exhibited significant changes in hemoglobin species. In addition, none of the clinical studies have yet shown an ability to use fNIRS at bedside for real-time monitoring. All the fNIRS data required later analysis.

Due to the inconsistent seizure detection rate, fNIRS has not been widely used in epilepsy clinical practice. This might reflect the fact that the theory of neurovascular coupling is a secondary phenomenon of primary neuronal hyperexcitability, which is subjected to further temporal delay on event capture. Therefore, targets other than hemoglobin species, such as CCO, may be more useful. CCO, as discussed earlier, is a mitochondrial complex that generates electrochemical potential, which drives ATP synthesis via ATP synthase. As the energy demand increases in hyperexcited neurons, intracellular ATP and corresponding oxidized CCO concentrations will change as well. In our institution, tens of patients have undergone simultaneous EEG and fNIRS monitoring with a custom-made device that is able to detect the change of hemoglobin species and CCO (Kadamati J. et al., 2018). This technology is well-tolerated, and there is no interference between the EEG and fNIRS. This pilot study sheds light on further understanding about metabolic and hemodynamic change during seizure. It potentially can offer an additional clinical parameter for more accurate earlier seizure detection and localization.

Vector diagram analysis to detect the initial dip in the hemodynamic response (Hong and Naseer, 2016), might also improve the temporal detection of seizure onset with fNIRS. However, the need for a higher sampling rate with fNIRS remains an obstacle, particularly for whole-head multi-channel systems. A new fNIRS device equipped with a sampling rate up to 100 Hz could result in comparable temporal resolution to EEG.

### Autonomic Functions

Other than assessing physiological changes of well-known cerebral phenomenon, fNIRS also can be a useful research tool for those diseases whose mechanisms are not fully explored. Autonomic dysfunction is one example. Our previous study has suggested that fNIRS can be utilized as a tool to assess autonomic dysfunction (Kadamati P. et al., 2018). A total of 12 subjects were recruited; 6 healthy controls, and 6 diagnosed with postural orthopedic tachycardia syndrome (POTS). Muscle oxygenation of lower extremities was measured via a commercial fNIRS device (INVOS 5100B) and custom-built optodes concomitantly. Expectedly, the subjects with POTS showed a higher degree of venous pooling (increased amount of total hemoglobin) in the lower extremities during 70-degree tilting. Furthermore, the responding time of the oxyhemoglobin and deoxyhemoglobin was faster in the healthy subjects than the POTS group, which suggests both neural reflexes and vascular compliance play roles in this group. These new findings from our study shed light on the mechanisms of POTS, and also of orthostatic intolerance in which patients do not have typical heart rate responses to tilting but have similar orthostatic symptoms.

Many other studies have also used fNIRS to investigate the cerebral hemodynamic dysregulation in patients with dysautonomia (Lankford et al., 2015), orthostatic intolerance (Tanaka et al., 2002) and vasovagal syncope (Ayers and Lawrence, 2015). For example, decreased temporal regional cerebral saturation was noticed to precede other changes of vital signs during vasovagal syncope (Ayers and Lawrence, 2015). Functional NIRS also yields a more reliable result for continuous cerebral blood flow. By analyzing the pattern of the dynamic change of cerebral blood flow, it is possible to expand the spectrum and the mechanism of orthostatic intolerance (Lankford et al., 2015). Therefore, fNIRS could potentially be a new biomarker for autonomic dysfunction. Moreover, diseases which are associated with cerebral hemodynamic dysregulation such as migraine can also be further studied with fNIRS.

### Migraine

A few groups have focused on the application of fNIRS to the detection of cortical spreading depression (CSD) in migraine. CSD is hypothesized as the main electrophysiological feature of migraine aura (see review (Goadsby et al., 2017)). In animal models, CSD is characterized by a short period of influx of sodium and calcium and intense depolarization of neurons and glias followed by a long-lasting “depression” of neuronal activity and decrease in regional cerebral blood flow (Lauritzen, 1994). Studying the regional oxygenation saturation and cerebral blood flow in migraine can provide more information about the mechanisms of migraine. Combining with TCD, in a self-controlled trial fNIRS has showed hypoperfusion secondary to decreased oxygen demand in the regions where aura and CSD presumably occur (Viola et al., 2010).

Several other groups have utilized fNIRS to evaluate cerebrovascular reactivity in patients with migraine. Delays in and reduced amplitude of peak oxyhemoglobin and total blood flow were observed in a patient with migraine during hypercapnia induced by breath-holding (Akin and Bilensoy, 2006). These findings suggest the possibility of impaired cerebrovascular reactivities in patients with migraine. Using different methods, similar conclusions were drawn by Shinoura and Yamada (2005). Specifically, transient positional intracranial hypertension resulted in less cerebrovascular reactivity in patients with migraine. Functional NIRS can be a potential biomarker to assess treatment responses to medication in patients with migraine. A more recent study has shown differences in hemodynamic changes between patients who received valproic acid, magnesium sulfate and dihydroergotamine suggesting their distinctive mechanisms for migraine treatment (Pourshoghi et al., 2015). In the next subsection, the potential of fNIRS as a diagnostic biomarker is further illustrated in mild cognitive impairment.

### Mild Cognitive Impairment

Mild cognitive impairment is characterized by cognitive decline that falls between that associated with normal aging and the more serious decline of dementia. It affects at least one of the cognitive domains, such as memory or complex thinking, but not activities of daily life. MCI is an etiologically heterogenous entity and about 15–20% of patients with MCI go on to develop clinical Alzheimer disease (AD) (Jessen et al., 2010). Neuropsychological testing has been the main biomarker used to distinguish patients with MCI, who eventually develop AD, from those with MCI who do not (Mueller et al., 2018; Meilan et al., 2020). Like other non-invasive neuroimaging modalities, fNIRS has been explored as a potential biomarker to differentiate MCI and AD (Hong and Yaqub, 2019). Recent fNIRS studies of MCI have shown a smaller increase in oxyhemoglobin (i.e., hypoactivation) in bilateral prefrontal lobes during working memory (Yeung et al., 2016; Vermeij et al., 2017) and in dorsolateral prefrontal cortices during memory retrieval (Uemura et al., 2016). However, the accuracy, on average 60–70%, has been too low to reliably identify MCI patients (Yang et al., 2019). Moreover, fNIRS has shown inconsistent results suggesting that patients with MCI have an increased oxyhemoglobin response in regions of interest compared with the healthy control group (Yoo and Hong, 2019). Therefore, the hemodynamic response detected by fNIRS is unlikely to be sensitive and specific enough for clinical practice.

More recently, the focus on non-invasive neuroimaging biomarkers for MCI and AD have shifted to functional connectivity. A recent systemic review including 36 articles over the past two decades suggests that patients with MCI and AD have impaired frontal and long-range connectivity in the resting state fMRI (Yeung and Chan, 2020), which was also observed in a recent study (Yoo and Hong, 2019). Task-related functional connectivity analysis suggests that the number and strength of prefrontal functional connections increased during a working memory task (Yu et al., 2020) but left and inter-hemispheric connectivity during a verbal fluency test were lower (Nguyen et al., 2019) in the patients with MCI compared to healthy controls. This could indicate the use of compensatory mechanisms in the prefrontal cortex as a result of impaired default connectivity in MCI patients. More research is needed to validate fNIRS against fMRI for use as a tool to characterize functional connectivity in patients with MCI.

## fMRI Validation of fNIRS

When it comes to widespread clinical use, such as for MCI, reproducibility (accuracy and precision) is a minimal requirement. It has been challenging to obtain consistent inter- and intra-subject results with continuous-wave fNIRS due to its relatively low SNR and the variability in scattering coefficients among subjects over time. Time- and frequency-domain fNIRS can be used to estimate the scattering coefficients in exchange of cost effectiveness and temporal resolution, making continuous-wave fNIRS more practical for use in a clinical setting.

Several studies have worked to validate fNIRS measures of oxy-, deoxy-, and total hemoglobin with fMRI, either simultaneously (Huppert et al., 2006; Steinbrink et al., 2006; Cui et al., 2011; Heinzel et al., 2013; Moriguchi et al., 2017; Wijeakumar et al., 2017) or separately (Okamoto et al., 2004; Maggioni et al., 2015). Using a cluster of voxels or a region of interest analysis, most fMRI studies have reported positive correlations (*r* > 0.4) between the BOLD response and fNIRS measures of deoxyhemoglobin (Huppert et al., 2006; Maggioni et al., 2015; Wijeakumar et al., 2017), while others have found stronger correlations between the BOLD response and fNIRS measures of oxyhemoglobin (Cui et al., 2011). The increased correlation between deoxyhemoglobin and the fMRI-BOLD response has been associated with the indirect measurement of task-dependent changes in deoxyhemoglobin due to metabolic demand (Huppert et al., 2006). Interestingly, Moriguchi et al. (2017) reported a stronger correlation in total hemoglobin (*r* = 0.33) compared to oxy- (*r* = 0.26) or deoxyhemoglobin (*r* = −0.2) using a bivariate correlation between fNIRS hemodynamic states and BOLD response during an n-back working memory task (Moriguchi et al., 2017). The reduced correlations across hemodynamic states could be related to task difficulty. Simple visual or motor based tasks have both shown increased correlations (*r* > 0.5) for oxy- and deoxyhemoglobin (Cui et al., 2011; Maggioni et al., 2015; Huppert, 2016; Wijeakumar et al., 2017). For example, during a finger tapping task with 14 fNIRS channels placed over the contralateral motor area, Huppert et al. (2006) reported a Pearson’s correlation of 0.71, 0.98, and 0.53 for zero-lag cross correlations between group averaged (*n* = 11) changes in the BOLD response to oxy-, deoxy-, and total hemoglobin respectfully.

While most validation studies have focused on group analysis, some have reported within subject results. In a finger tapping experiment, Huppert et al. (2006) showed similar correlations between hemoglobin concentrations and the BOLD response on an individual subject level. During a visual task, Maggioni et al. (2015) report correlations between oxy-, deoxy-, and total hemoglobin concentrations and the BOLD response measured within a 1 cm spherical radius projected below the fNIRS channel for 8 subjects (Maggioni et al., 2015). For deoxyhemoglobin, task-based correlations ranged from 0.26 to 0.83 across subjects. The high inter-subject variability could help explain why more complicated tasks, such as working memory, show decreased correlations in group analyses (Cooper et al., 1997; Huppert et al., 2006; Cui et al., 2011; Maggioni et al., 2015).

Techniques that account for subject-specific differences in anatomy and sensor positioning can improve the correspondence between fNIRS and fMRI measures of task related activity within subject. The impact of subject variability can be reduced by projecting fNIRS channel data onto subject specific anatomy. This has recently been done by Wijeakumar et al. (2017) who showed that the mapping of fNIRS channel data onto subject specific anatomy and projection from the fNIRS channel space to the fMRI voxel space improved the correlation between fNIRS and fMRI measures of task activity. Using a voxel cluster correlation, they reported correlations ranging from 0.62 to 0.99 for both oxy- and deoxyhemoglobin.

## Limitations of fNIRS and Future Directions

In summary, fNIRS is an appealing technology in the field of neuroscience due to its portability, tolerability, non-invasiveness, cost effectiveness, and ability for long-term real-time monitoring. However, fNIRS has, so far, had limited clinical use due to the lack of anatomic specificity, suboptimal temporal resolution, and low intra-subject reproducibility for individual analysis. The spatial resolution of fNIRS is also less than that of fMRI. Whereas fMRI provides homogeneous resolutions of 1–3 mm, the spatial resolution of fNIRS is limited by the number of detector optodes and the physics of light scatter from the emitter to the detector. Current systems uses detectors spaced ∼3 cm apart from an illuminating optode that inform on brain activity along a curved path from the emitter to the detector, increasing the spatial uncertainty of the signal source both laterally (between the emitter/detector pair) and in depth.

Increasing optode density, such as multichannel fNIRS, can greatly improve the anatomic specificity. Multichannel has been widely used increasingly in research (Hong and Yaqub, 2019). However, multichannel fNIRS optode caps can become uncomfortable to wear over time, due to the weight of the cables and the pressure of the optodes on the subject’s scalp. Reducing cable weight by using an armature can help reduce discomfort for prolonged recordings. In addition to the tolerability concern, multichannel fNIRS could impact concurrent EEG monitoring. Hybrid EEG-fNIRS caps are designed to tackle this problem (Hong and Khan, 2017). However, in many clinic settings including pediatric populations and for patients with cranial defects or abnormalities, the hybrid cap with fixed electrode placement can dramatically decrease the accuracy of EEG.

An additional limitation of fNIRS is the lack of access to cortical regions not adjacent to the scalp. The ventral surface of the frontal cortex, the basal ganglia and much of the cerebellum cannot be readily measured with fNIRS due to the increased light absorption with distance from the scalp. Variations in the thickness of the skull and adjacent tissues can affect the inter-subject sensitivity of fNIRS, especially in adults. Time-domain and frequency-domain fNIRS can increase depth sensitivity (Lange et al., 2019) and have proved to be reliable for detecting oxygenation in deep tissue at the cost of sampling rate, instrument cost effectiveness and computational tolerability. At present, optimum processing of fNIRS signals requires advanced knowledge of computer science, signaling processing, and engineering, which can reduce the perceived accessibility of the fNIRS for use in the clinical environment.

Another way to increase the anatomic specificity is through co-registration of fNIRS sensor locations to individual subject’s anatomy (via MRI). If anatomical MR scans are available, the use of subject specific parameters, such as partial path length, skull thickness, and skull curvature could be measured and used to account for individual differences in the forward model of fNIRS signal propagation. For patients with neurologic dysfunction, full head imaging could prove especially important for spatial localization and identification of eloquent cortex before surgical intervention.

Compared with EEG, the lower temporal resolution of fNIRS restricts its clinical application. The physiological window for hemodynamic change occurs over seconds while the electrical changes occur over tens to hundreds of milliseconds. Minimally sampling rates of 100 Hz are required for EEG, and most clinical laboratories sample at more than 512 Hz. Commonly used sampling rates for fNIRS are in the tens of Hertz and are even lower for whole-head multi-channel systems. Newer machines using higher sampling rates of ∼100 Hz would provide comparable temporal resolution to clinical EEG. Functional NIRS with higher sampling rates could be particularly useful for CCO detection since it directly responds to increases in cellular metabolism secondary to neuronal activity.

The intrasubject reproducibility is determined by a variety of environmental factors including the presence of ambient light, skull thickness, hair density and color (dense dark hair can block light transmission and reduce signal strength), and head motion. For some factors, experimental preparation to reduce ambient light and separate hair follicles during sensor placement can mitigate their impact prior to data collection, while others, such as head motion, can often be corrected during post-processing by leveraging techniques developed for fMRI and EEG analyses.

Recent advances in neural network learning has increased the accuracy of fNIRS (Yang et al., 2019) and could improve its utility for online monitoring of patients. Processing techniques, such as adaptive filtering, can be used online to provide real-time feedback on neurological conditions (Izzetoglu et al., 2010; Nguyen et al., 2018) but will require computational refinement for online processing during full head imaging.

**FIGURE 4:**
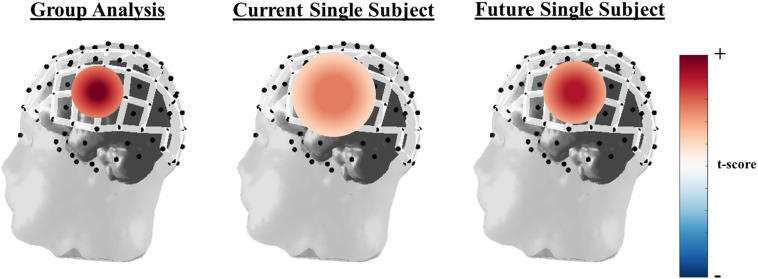
Idealized GLM output for a right-hand finger tapping task at the group **(left)**, current single subject fNIRS analysis capabilities **(middle)**, and where single subject fNIRS analysis needs to be in order to be clinically relevant **(right)**. The example of oxyhemoglobin is used in this depiction.

Finally, fNIRS studies report results at the group level to increase statistical power. While this approach enables the use of fNIRS for neuroimaging research into brain function, its limits the clinical utility for individual diagnosis. In order for fNIRS to be used in clinical assessment of brain function, such as identifying language lateralization in epileptic patients before surgical resection, consistent and robust results need to be obtained at the individual level. An idealized general linear model (GLM) example of the differences in group versus individual subject level analysis can be seen in Figure 4. Here the depiction shows strong statistical increase in t-scores over a specific set of channels known to be active during a right hand finger tapping task at the group level, while the current capabilities of individual subject analysis shows a more broad region with a smaller increase in t-scores. In order to be more clinically relevant, advanced processing and hardware configurations, such as those mentioned in this review, are critical. Further developments of the field are needed to identify a more spatially localized and significant result then what is currently being done. The use of high-density whole head optode arrays, precise sensor locations relative to the head, co-registration with anatomic scans, short-distance channels, and improvements in signal processing can be combined to improve within subject analyses.

## Author Contributions

All authors contributed extensively to the work presented in this manuscript. W-LC and JW wrote the first draft of the manuscript. JW, NH, JS, and SB wrote additional sections of the manuscript. All authors reviewed and edited the manuscript.

## Conflict of Interest

The authors declare that the research was conducted in the absence of any commercial or financial relationships that could be construed as a potential conflict of interest.
